# Faculty Development for Small-Group-Teaching with Simulated Patients (SP) – Design and Evaluation of a Competency-based Workshop

**DOI:** 10.3205/zma001119

**Published:** 2017-10-16

**Authors:** Henrike Hölzer, Julia Freytag, Ulrike Sonntag

**Affiliations:** 1 Medizinischen Hochschule Brandenburg Theodor Fontane, Neuruppin, Germany; 2 Charité-Universitätsmedizin Berlin, Abteilung für Curriculumsorganisation, Berlin, Germany

**Keywords:** didactics, faculty training, simulated patient, training concept, competence

## Abstract

**Objective: **The introduction of innovative teaching formats and methods in medical education requires a specific didactic training for teachers to use complicated formats effectively. This paper describes preliminary considerations, design, implementation and evaluation of a skills-based workshop (7,5 hours long) for teaching with simulated patients. The aim is to describe the essential components for a lasting effect of the workshop so that the concept can be adapted to other contexts.

**Method: **We present the theoretical framework, the objectives, the didactic methodology and the implementation of the workshop. The evaluation of the workshop was carried out using questionnaires. First the participants (teachers of the faculty of medicine, clinical and science subjects) were asked to estimate how well they felt prepared for small group teaching immediately after workshop. Later, after some teaching experience of their own, they gave feedback again as a part of the general evaluation of the semester.

**Results:** In the course of three years 27 trainings were conducted and evaluated with a total of 275 participants. In the context of semester evaluation 452 questionnaires were evaluated on the quality of training.

**Conclusion:** The evaluation shows that participants appreciate the concept of the workshop and also feel sufficiently well prepared. As a limitation it must be said that this is so far only the lecturers’ self-assessment. Nevertheless, it can be stated that even a one-day workshop with a stringent teaching concept shows long term results regarding innovative teaching methods.

## Zusammenfassung

**Zielsetzung: **Die zunehmende Einführung innovativer Lehrformate und -methoden in die medizinische Ausbildung macht eine spezifische didaktische Schulung der Lehrenden erforderlich, um aufwendige Formate effektiv nutzen zu können. Der vorliegende Beitrag beschreibt Vorüberlegungen, Konzeption, Ablauf und Evaluation eines kompetenzbasierten Workshops (10 Unterrichtseinheiten) zum Unterrichten mit Simulationspatienten. Das Ziel ist, die für eine nachhaltige Wirkung wesentlichen Bestandteile des Workshops so zu beschreiben, dass das Konzept für den eigenen Kontext adaptiert werden kann.

**Methodik:** Es werden die zugrunde liegende Theorie, die Ziele, die didaktische Methodik und die Umsetzung des Workshops vorgestellt. Die Evaluation des Workshops erfolgte mittels Fragebögen. Die Dozierenden der Humanmedizin (klinische und theoretische Fächer) schätzten zunächst unmittelbar nach der Schulung ein, wie gut sie sich vorbereitet fühlen. Später, nach eigener Unterrichtserfahrung, gaben sie darüber im Kontext der Semesterevaluation erneut Rückmeldung.

**Ergebnisse:** Im Verlauf von drei Jahren wurden 27 Schulungen mit insgesamt 275 Teilnehmenden durchgeführt und evaluiert. Im Rahmen der Semesterevaluation wurden 452 Fragebögen zur Qualität der Fortbildung ausgewertet.

**Schlussfolgerung: **Die Evaluation zeigt, dass die Teilnehmenden das Konzept des Workshops schätzen und sich auch längerfristig ausreichend gut vorbereitet fühlen. Einschränkend ist zu sagen, dass es sich dabei bislang nur um die Selbsteinschätzung der Dozierenden handelt. Dennoch kann festgehalten werden, dass auch ein eintägiger Workshop mit einem stringenten Unterrichtskonzept zur Qualitätssicherung der Lehre mit innovativen didaktischen Methoden beiträgt.

## 1. Introduction

We know that teachers need to be trained in using appropriate didactic methods to be able to carry out their tasks in medical education effectively [1], [2]. To this end, workshops are often the method of choice, because they need few resources: human resources and planning costs are low and the participants will save time and costs as well [3]. On the other hand the usefulness of workshops is controversial because one workshop as a single event can hardly cause sustainable results [4]. So far there are few comprehensible descriptions of successfully evaluated training models [5]. Our study helps to fill this gap. We show how a single workshop about the employment of a specific didactic method, namely simulated patients (SP), can address this tension between efficiency and sustainability. It is important to convey general skills that may be applied to other events and content, while at the same time providing specific methodological support, which the lecturers can use immediately.

The concept, the execution and the evaluation of a workshop will be described. This workshop specifically deals with the question of how lecturers employ SP for small group teaching with the highest possible benefit. It is accompanied by highly structured manuals and online teaching materials. Optionally the lecturers may also attend in-depth training on various techniques of medical communication.

## 2. Project Description: Workshop “Working with simulated patients”

The aim of teacher trainings at the university is to support the lecturers in the professionalization of their activities and ensure that they have or can acquire the necessary expertise [6]. Innovative teaching methods that few lecturers already know from their own training, such as the use of SP in the classroom, require special training to exploit their potential.

At the Charité SP have been used in the classroom [7] since the year 2000. After the new model curriculum had been introduced in 2010, SP performed nearly 2000 times annually in the small-group-teaching format called KIT (an acronym for communication, interaction, teamwork). KIT is a longitudinal seminar, which stretches over eight semesters in the curriculum of human medicine. All students experience 25 different SP scenarios. The SP scenarios serve to guide the students how to use interviewing techniques such as motivational interviewing [8] or models such as NURSE [9], [10]. In addition, social skills such as a careful approach to delicate issues or intercultural sensitivity are practiced. For lecturers, this means that they are faced with a new weighting of teaching content, a new teaching format and unfamiliar teaching methods. What is more, they are also confronted with a new organizational structure, because format and content are defined centrally and thus across institutions.

Clinical duties allow few faculty members to participate in extensive faculty development programs. The workshop-format allows the preparation of a large number of people with the required minimum qualification. Since August 2012, the scope of the preparatory training for new teachers of KIT has been increased from 4 to 15 lessons. Five units of which are used to prepare the topics of exactly that semester, in which the lecturers plan to teach. Ten Lessons, which may be completed either in one day or on two consecutive half-days, primarily serve to learn how to effectively apply the method "Simulated Patient". A training group usually consists of 10-12 lecturers, who belong to different disciplines – even basic sciences like physiology and anatomy. Attendance is mandatory for all faculty who are teaching formats that work with SP. 

### 2.1. Preliminary considerations

The term competency describes a person’s ability to do the right thing in a specific context, i.e. a combination of knowledge, skills and attitude [11], [12]. Teaching competency does not only include subject expertise, but also a learner-centered approach, social and communication skills, professional behavior – also as a role model – as well as reflective practice and a systems-related practice. Specific qualifications on the micro level are also required [6], concerning the tools, the teachers need for their courses. Even more so, as simulated patients are used for different purposes and with a different approach at different locations [13]. When designing faculty development workshops, it is therefore essential to take into account the needs of individual lecturers and students and also institutional requirements.

#### 2.2 Learning objectives of the workshop

The workshop concept was based on the "Professional Standards for Medical Educators" [http://www.medicaleducators.org/index.cfm/profession/professional-standards/]. Although the recommendation is primarily aimed at supervisors rather than teachers themselves, the values and domains described therein are also relevant to the user level. In the second domain which outlines standards for “Teaching & facilitating learning” [http://www.medicaleducators.org/write/MediaManager/Documents/Teaching-and-_facilitating-learning.pdf], six key ingredients are described:

1. delivering teaching,
2. maintaining an effective learning environment,
3. learning and teaching methods and resources,
4. feedback on learning,
5. Ensures active participation and learner engagement and finally
6. reflection.

Whereas the Academy of Medical Educators specifies the requirements in general for all teaching formats, we look at them with the complex teaching situation of simulation in mind. The teachers have no other opportunity to prepare practically for their task but in this workshop. There the participants are introduced to the concept of the teaching format, learn to create a safe atmosphere, learn about the teaching method SP and how to apply it, learn to guide reflection, and to actively involve learners. The specific learning objectives pursued by the workshop are shown in Table 1 (Tab. 1) below.

It is only possible to take into account those six aspects in the context of a single workshop, if the focus is limited to one teaching format (simulation in small group instruction) with clearly defined content (social and communicative skills with defined learning objectives). Other topics, such as group dynamics or self-reflection are addressed, as they are both crucial for the implementation and evaluation of SP-talks. The overall goal is to support the lecturers in integrating the SP into their teaching with as much benefit as possible to maximize learning.

#### 2.3 Structure and schedule of workshops

Theories of adult education recommend establishing previous knowledge and experience of participants at the beginning of an intervention [14]. Therefore we start with a brainstorming session on possible methods of teaching social and communicative competences. Next, results of medical education research on teaching communication skills are being presented as a short overview as well as the communication curriculum at the Charité. Several studies recommend doing so to convince the participants of the relevance of the teaching objectives [15], [16], [17]. This is followed by a detailed description of the qualifications and capabilities of SP in general and specifically at the Charité. The advantages and disadvantages of the method SP are contrasted with other educational approaches such as role-play among students or bedside teaching. Based on an exemplary KIT session we demonstrate how to interact with the SP in the classroom. A flow chart has been created to demonstrate the preparation, implementation and evaluation of SP-talks including feedback, which is also known to the SP. The sequence of a typical SP-interaction will be explained in the workshop with posters that the lecturers will also find in their manuals (see Attachment 1). The next item is a contribution from a student special interest group KIT, which clarifies what the students expect from this format of instruction. Furthermore the participants reflect about feedback rules and practice how to give feedback themselves on a video example of an interaction between a SP and a physician. There is a wealth of evidence (among others [1], [18], [19]) on the effectiveness of systematic, constructive feedback. Thus it is indispensable in medical education today.

Finally, the first of two live simulations of lessons will take place. One participant takes on the role of the teacher while all other participants take on the role of students. The teacher’s task is to prepare the SP-contact together with the group after the previously developed scheme and evaluate the interaction according to the standards. For this purpose, a less complex SP-scenario is used. Following the 60-minute simulation of the teaching situation, the teacher’s job will be evaluated. Initially the lecturer reflects about his/her teaching experience, afterwards he/she receives feedback from the other participants and the trainer. Finally, the lecturer him/herself draws a conclusion. In the second simulation, a more sophisticated SP scenario is used and evaluated as described above.

The last part of the course is devoted to the group dynamics of teaching in small groups. The participants reflect on their own teaching experiences, to develop theoretical foundations such as roles and functions in groups, stages of group processes, etc. Moreover, they can try out different exercises they will later use in class. This unit is focused on the role of the teacher and the corresponding tasks and opportunities for intervention. The event is called to a close by a joint verbal evaluation. 

Timing and sequence of training are shown in Table 2 (Tab. 2) below.

#### 2.4 Teaching Methodology

The workshop about teaching with SP is constructed so that the lecturers can experience or try out themselves much of what they will use later in the classroom. Other success factors are the variable use of different methods, and the possibility for exchange with colleagues as well as to give and receive collegial support [1]. So they will not only receive practical tips (e.g. about what to do when no one volunteers) but they will also continually be offered opportunities for reflection in the group. The majority of participants have no experience with SP, so it is important to inform them that strong emotions can be triggered by the simulation. It is not sufficient only to practice an interview technique "on object" or to just "survive" the situation to achieve a learning effect. The applied skills must be reflected to generate a growth of knowledge and be transferred into practice. Following Kolb's theory of experiential learning [20] concrete experiences are the basis of observations and reflections, which in turn form the basis for abstract concepts whose practicality can then be retested [21]. Therefore, in the interactions with SP, there is special emphasis put on the lecturer’s tasks of relating the experience to the learning objectives, guiding reflections and securing the results.

If the lecturers themselves are exposed to the same situation that they demand from students, they will realize, first, that a simulated situation is not transferable to reality 1: 1 (as a singular situation is not representative of the learner’s competence) and second that there is a fundamental difference between the experience of observers and the experiences of the participants [22]. This experience should help to ensure that the lecturers recognize the importance of a safe learning environment for students and consequently create a learning environment with a clear division of roles and tasks, which provides orientation [23], [24].

## 3. Method

Immediately after the training the participants filled out an evaluation sheet with eight items on a five-point Likert scale (1=strongly agree – 5=strongly disagree) and four open questions. The four open questions were based on what the participants particularly liked or disliked about the event, what suggestions they have for this workshop and what training opportunities they would want in the future. The evaluation should tell us, if the participants have achieved the learning outcomes in their own opinion (in particular the skills), whether they found the content relevant and whether they were satisfied with the structure and the methods of the event in general.

In addition, all KIT lecturers were interviewed with a further questionnaire at the end of the semester. Unlike directly after the training, the lecturers can now relate the value of the training to the requirements of teaching practice. For the period from summer semester 2013 to winter semester 14/15 (with a return rate of an average of 47% of all lecturers teaching in one semester) a total of 452 questionnaires were used for the re-evaluation. The high number of cases comes about because the lecturers evaluate again after each semester and therefore some of them were questioned repeatedly. In the questionnaire for the re-evaluation two questions are included asking about qualification offers. On a scale of “1=strongly agree” to “5=strongly disagree” the lecturers answered the item: “I felt absolutely well prepared for the teaching activity by the teacher-training”. The second question is “Would you like further opportunities for training to improve your teaching activities?”. A pre-test of these items was not carried out, because the questions were part of already established instruments for quality assurance of didactic trainings of the faculty. All data were analyzed using SPSS, version 22nd. Averages and standard deviations were calculated. The content of the free text comments was categorized and evaluated.

## 4. Results

In the period from August 2012 to August 2015 27 training sessions were held, a total of 275 lecturers participated. The number of participants per training ranged between 5 and 17. On average, 10 people participated. The training was carried out by three different trainers who have several years of experience with the employment of SPs in teaching.

### 4.1. Evaluation immediately after training

Table 3 (Tab. 3) shows the results of the individual items of the evaluation directly after the training.

90% (n=248) of the participants evaluated the workshop using free-text comments. One third praised the high level of practical exercises and the ideal balance between theory and practice. Some practical exercises were highlighted as particularly helpful. The participants praised in addition, the variety of used methods, the supportive learning environment and the constructive atmosphere. The most critical issue was the duration: some participants found the workshop to be too long or thought the time could be used better. Suggestions for improvement were even more practical exercises (e.g. more SPs), the demonstration ideal examples (live or video) or reports by experienced lecturers. The participants see further training needs especially in the areas of exchange of experience/feedback (peer intervision or supervision) and they desired refresher workshops. A fifth of respondents want training for special educational topics, such as for dealing with difficult students.

#### 4.2. Renewed evaluation after participants’ own teaching experience

Respondents felt well prepared by the teacher-training for their teaching activities (95% valid feedback with a mean of 2.3 [SD 0.87]). 55% gave an affirmative answer to the question “Would you like further opportunities for training to improve your teaching activities?”.

## 5. Discussion

The workshop is designed as a basic workshop. It is often the first university teaching training for the participants. The high consent to questions 2 and 3 of the evaluation immediately following the training shows that the participants trust the skills they acquired. While on the Four-Level Model of Evaluation developed by Kirkpatrick [25] the learner’s self-evaluation is only on level 1 “reaction” and 2 (subjective) “learning outcomes”, the great satisfaction of the participants may be seen as an indicator of high self-efficacy and high motivation. Both are according to Bandura [26] important prerequisites for successful implementation of what has been learned. The content of the training is perceived as relevant, which is shown by the approval of the first item. With still good values, the question of “group dynamics” is more critical. Probably, the participants could still be better prepared for “typical critical situations” or maybe even an extra training on this subject is required. This view is supported by the participants’ desire for training on dealing with “difficult students”, which was repeatedly expressed. 

In the literature, a high amount of practical content is required, as well as the possibility to reflect and learn from their own experiences [27]. The answers to items 5-7 and the majority of free text comments shows that this is exactly what the participants value the most. Of particular importance are the issues *learning environment, work atmosphere and exchange with colleagues*.

The evaluation of all KIT lecturers shows that participants in retrospect feel rather well prepared for the “practical test” by the training. While they still perceive some deficits, those might as well be related to the content area – in the sense of the specific topics of KIT-courses in the different semesters – and do not necessarily refer to the method “Simulation Patient”, to which the training described here is aimed.

From a methodological perspective it should also be noted that the questionnaires of the later survey are unfortunately not attributable due to the anonymity of the data collection. Thus, it must be conceded that in the second evaluation – as already mentioned – the same persons were probably asked several times. It is also possible that the training was attended longer ago in individual cases. Nevertheless, there is a clear trend to a positive assessment of the training.

It can be discussed whether the lecturers would have felt well prepared even without any training. However, the experiences of the course instructors and the students speak to the contrary. Although attendance of the workshop is mandatory, it still happens that individual lecturers, e.g. when substituting, teach without this preparation. When that happens, student evaluation shows critical feedback with the demand of appropriate qualifications of the lecturers. Also the workshop-part “Student perspective on KIT” has been included on the explicit request of students for qualified lecturers and follows students’ need to participate in it.

If participants are prompted to name additional training requirements, there is no clear trend. On one hand, refresher workshops and more possibilities for peer exchange are required (intervision or supervision), on the other hand, the training opportunities are described as adequate. Possibly this indicates a conflict between the qualification requirements and the conditions that often make it difficult for lecturers to participate in training and educational programs at all. This interpretation might be further supported by comments about time and duration of the event (e. g. “workshops in the future, please only after 4 p.m.”).

## 6. Conclusion

In an increasing number of medical schools new teaching formats are used, resulting in a changed need for training for effective use of innovative teaching methods. Our study shows that a workshop “just-in-time” [28] is a useful qualification for the preparation of lecturers for a new teaching format. We hope to show, as required by Bylund [5], how lecturers can be prepared methodically within a manageable time frame on teaching communication and social skills. Our example is not at all limited to local characteristics, but can also be transferred to other departments.

While we have now shown how the workshop affected the self-assessment of the lecturers, it still remains open to what extent the lecturers actually implement the standards of the SP program in the classroom. This is investigated in a recent observational study with lecturers, who were trained with this concept. A preliminary analysis of the results indicates that the majority of the standards will be implemented by the lecturers, so that the self-assessment of the lecturers seems to be concordant with the assessment of the observers.

## 7. Acknowledgments

We thank Rita Kraft for assistance with data collection and analysis as well as Rolf Kienle, Isabel Mühlinghaus and Simone Scheffer, who elaborated with us the execution of SP-talks.

## Competing interests

The authors declare that they have no competing interests. 

## Supplementary Material

2 Poster: • Preparation of SP-talks • Analysis of SP-talks

## Figures and Tables

**Table 1:**
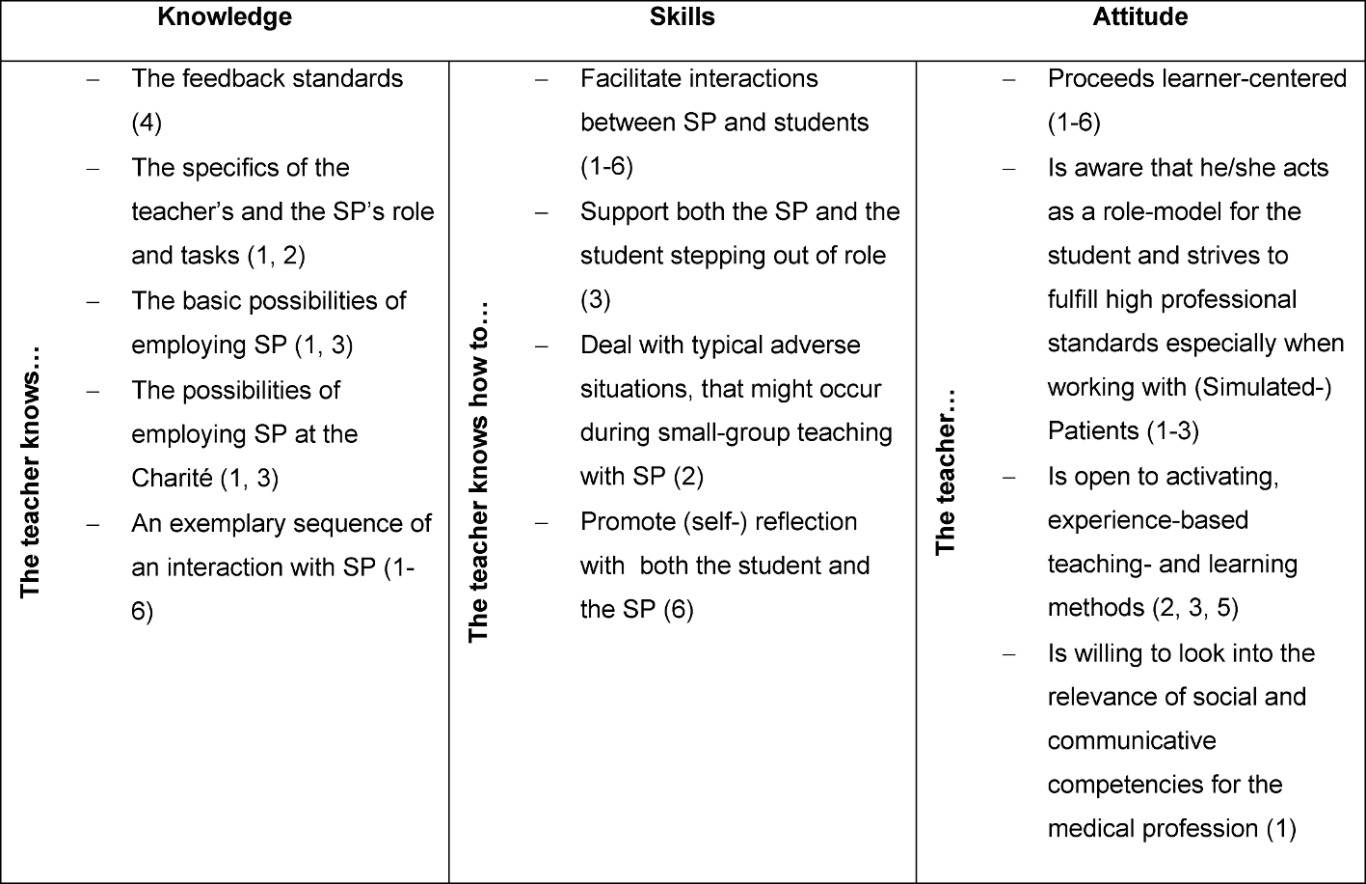
The workshop’s learning objectives and how they relate to the pursued competencies

**Table 2:**
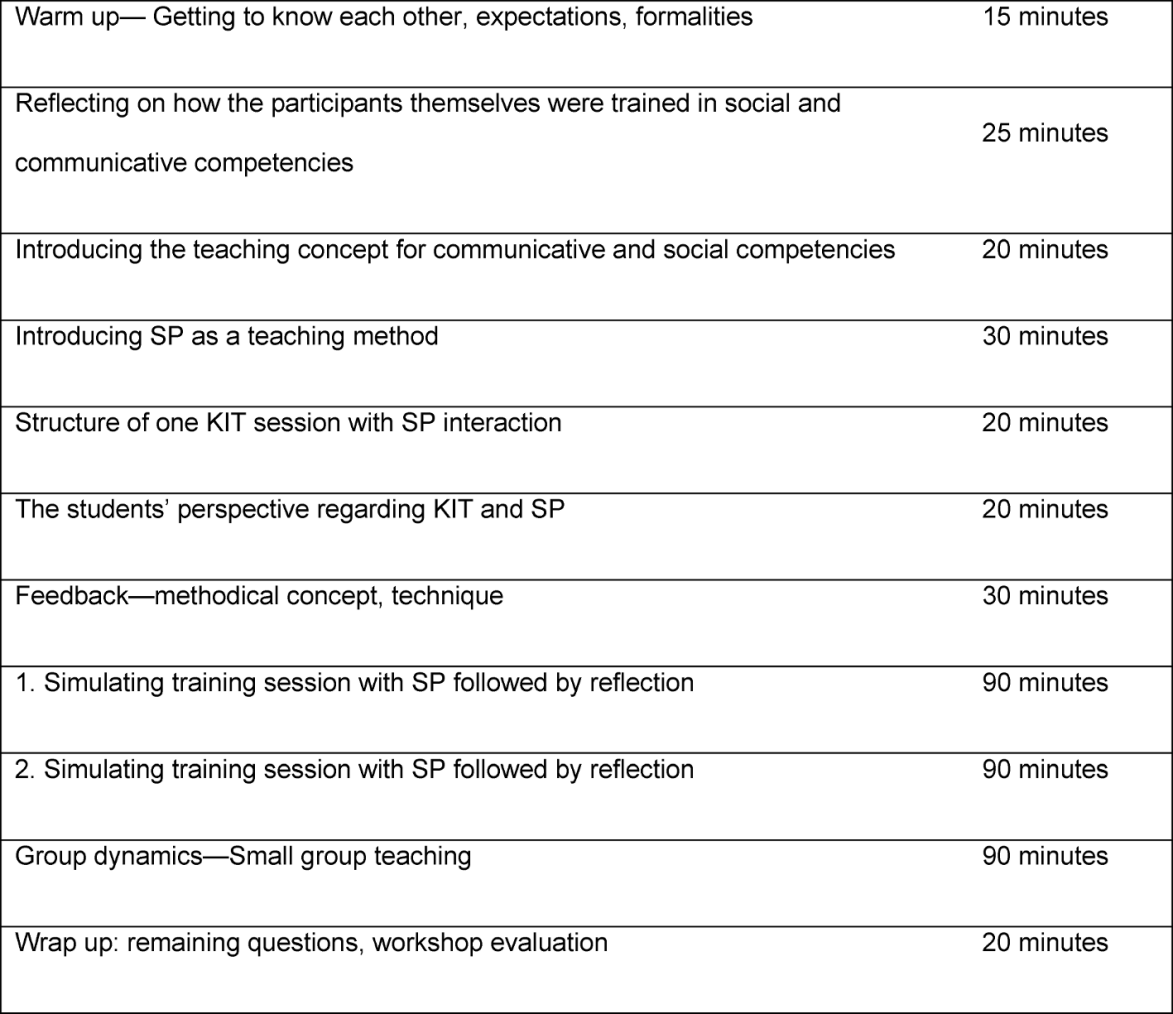
Structure of the teacher training

**Table 3:**
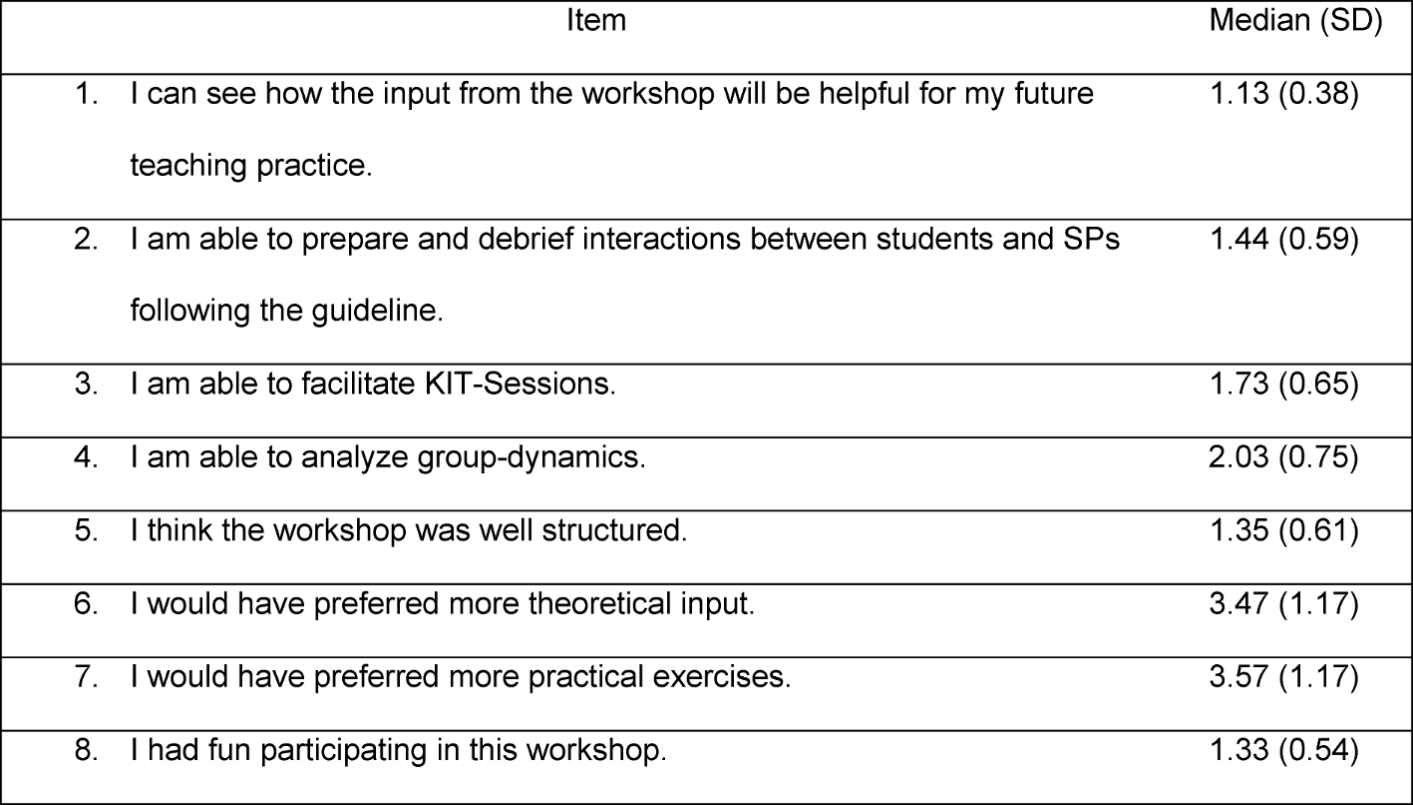
Median and standard deviation (SD) of the workshop evaluation (1=agree completely – 5=do not agree at all; N=275 for 27 workshops)
