# Supplementary material for: Faculty Development for Small-Group-Teaching with Simulated Patients (SP) – Design and Evaluation of a Competency-based Workshop
Source: GMS J Med Educ. 2017 Oct 16;34(4):Doc42. doi: 10.3205/zma001119 (PMC5654117; doi:10.3205/zma001119)
Supplement: 2 Poster: • Preparation of SP-talks • Analysis of SP-talks [file JME-34-42-s-001.pdf]

Vorbereitung von SP-Gesprächen

Ablauf

1. In der Gruppe klären, wer das Gespräch führen wird.

2. Der Gruppe Vorinformationen über die Gesprächssituation geben.

3. In der Gruppe Lernziele besprechen und Vorwissen aktivieren.

4. Beobachtungsaufgaben in der Gruppe verteilen.

5. Das Setting für das Gespräch gestalten.

6. Den/die SP kurz über das Setting informieren.

Bitte beachten

- Das Gespräch beginnt und endet im Raum.
- Sobald das Gespräch beginnt, ist die Dozentin/der Dozent in einer Beobachterrolle und unterbricht nicht.

Auswertung von SP-Gesprächen

Ablauf

1. Der/Die SP verlässt den Raum, um das Feedback vorzubereiten.

2. Alle Beobachter überlegen und strukturieren ihr Feedback im Stillen.

3. Dozent/in und Gruppe gestalten das Setting.

4. Die Auswertung des Gesprächs beginnt erst, wenn die/der SP wieder im Raum ist.

5. Dozent/in moderiert das Feedback:

- Lernende/r äußert sich
- SP gibt Feedback
- Gruppe gibt Feedback
- Dozent/in gibt Feedback

6. Reflexion. Dozent/in leitet die Diskussion der Gruppe und sichert die Ergebnisse

7. Der/Die SP wird verabschiedet
